# Association between albumin infusion and sepsis risk of patients with acute pancreatitis

**DOI:** 10.1371/journal.pone.0314738

**Published:** 2025-08-07

**Authors:** Xuan Zhou, Bangyan Jiang, Ningjing You, Xinfeng Qian, Dong Han, Wei Sun

**Affiliations:** Department of Emergency, Affiliated Hospital of Jiangnan University, Wuxi, Jiangsu Province, China; Yale University, UNITED STATES OF AMERICA

## Abstract

**Objective:**

To investigate whether early administration of serum albumin infusion in the acute pancreatitis (AP) patients admitted to the intensive care unit (ICU) reduces the risk of sepsis.

**Methods:**

Data were collected from the Medical Information Mart for Intensive Care III and IV databases for this retrospective cohort study. The primary outcome was the occurrence of sepsis in AP patients. We used univariate and multivariate logistic regression models to evaluate the association between albumin infusion and sepsis risk of AP patients. Additional subgroup analysis by stratification to serum albumin were also performed.

**Results:**

The study included 779 patients with AP. They were divided into a sepsis group (83 patients) and a non-sepsis group (696 patients) according to whether they developed sepsis, and the prevalence of sepsis was approximately 10.65%. Multivariate logistic regression model indicated that albumin infusion was associated with decreased risk of sepsis in the AP patients [adjusted odds ratio (OR)=0.37, 95% confidence interval (CI)=0.13–0.88]. Subgroup analysis showed a negative association between the albumin infusion and sepsis risk in the AP patients with serum albumin ≤ 3.5g/L (adjusted OR=0.29, 95%CI = 0.08–0.77).

**Conclusion:**

In this study, we found an association between albumin administration and a lower risk of sepsis in AP patients, which persisted after multivariate adjustment. This suggests that albumin infusion may have unique potential benefits for this population.

## Introduction

Acute pancreatitis (AP) is a prevalent inflammatory disorder affecting the pancreas, characterized by persistent, acute abdominal pain of varying severity [1]. It is reported that the incidence of AP worldwide is 33 cases per 100,000 person-years and exhibits a gradually increasing trend in occurrence [2,3]. Sepsis, caused by a dysregulated host response to infection, is a common complication in the intensive care unit (ICU) that ultimately leads to septic shock and multiple organ failure [4]. In the course of AP, early concurrent infections (such as pneumonia or bacteremia) and subsequent secondary infections of the pancreas or peripancreatic necrosis may lead to the onset of sepsis [5,6]. The incidence of secondary pancreatic infection and sepsis in AP cases ranges from 40% to 70%, significantly increasing the mortality rates for these specific patients [7]. Therefore, identifying the factors associated with reducing the risk of developing sepsis for AP patients holds significant clinical implications.

Serum albumin is widely recognized as a crucial biomarker in predicting adverse outcomes in AP, particularly for the prediction of persistent organ failure and mortality [8,9]. Among AP patients with hypoalbuminemia, there were significant amounts of non-esterified fatty acids that are not bound to albumin, leading to impaired immune cell function and an increased risk of infection and sepsis [10]. Hypoalbuminemia was also found to be associated with the acquisition and severity of infectious diseases [11]. These findings suggested that hypoalbuminemia may play a significant role in the complications of sepsis in patients with AP. In recent years, albumin infusion has increasingly become a prevalent method for treating hypoproteinemia in critically ill patients [12]. A retrospective study showed that albumin infusion was associated with improved 28-day mortality among patients with septic shock [13]. Compared to saline, albumin administration did not impair renal function and potentially reduced the risk of mortality [14]. In the study of Xu et al., they concluded that albumin infusions may significantly reduce mortality in hypoalbuminemia patients with severe acute pancreatitis [15]. However, few studies have investigated the efficacy of albumin infusion in the complications of sepsis in patients with AP.

Thus, the objective of this study was to investigate whether early administration of serum albumin infusion in the AP patients admitted to the ICU reduces the risk of sepsis, providing valuable insights for guiding the treatment strategy for AP patients.

## Methods

### Data sources and study population

This study was based on open intensive care clinical databases, known as Medical Information Mart for Intensive Care (MIMIC-III and MIMIC-IV). MIMIC-III is a freely accessible database that comprises information from over 40,000 individuals who were admitted to the ICU at Beth Israel Deaconess Medical Center between 2001 and 2012 [16]. MIMIC-IV database, an updated version of MIMIC-III, contains the medical records of all patients who were admitted to the ICU at Beth Israel Deaconess Medical Center from 2008 to 2019 [17]. Because the MIMIC-III and MIMIC-IV databases are publicly available anonymized databases, approval from the ethical committee of Affiliated Hospital of Jiangnan University was not necessary.

**Fig 1:**
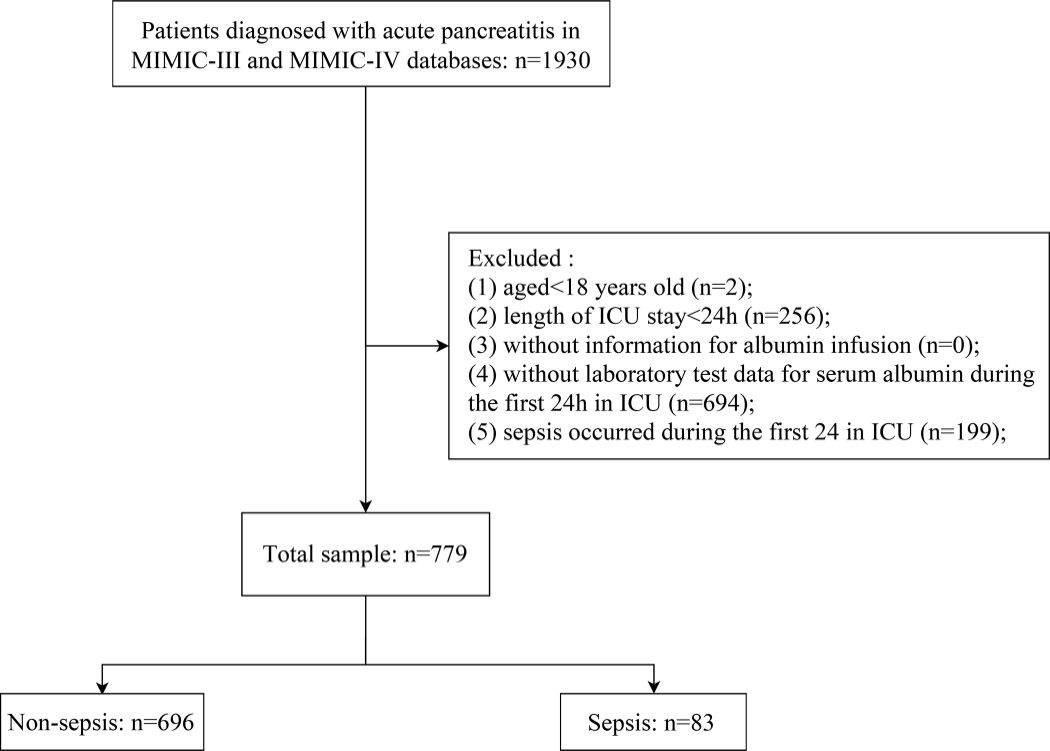
Flowchart of study patients.

The criteria for selecting the subjects were the following: patients diagnosed with AP, who were selected according to according to International Classification of Diseases, 9th Revision (ICD-9) code 577.0 and International Classification of Diseases, 10th Revision (ICD-10) codes K85-K85.92. Patients who meet the following criteria were excluded: (1) patients younger than 18 years of age; (2) patients who stayed in the ICU for less than 24 h; (3) patients without information for albumin infusion; (4) patients without laboratory test data for serum albumin during the first 24h; (5) sepsis occurred during the first 24h in ICU; (6) patients who developed sepsis within 24h of ICU admission and readmitted patients. MIMIC-III included 325 patients, and MIMIC-IV included 454 patients, Ultimately, 779 patients were enrolled in this study (after applying the exclusion criteria) (Fig 1).

### Outcome

The primary outcome was the occurrence of sepsis in AP patients. Sepsis was diagnosed according to the sepsis-3 criteria: patients with documented or suspected infection and an acute change in total Sequential Organ Failure Assessment (SOFA) score of ≥ 2 points [18].

### Assessment of albumin infusion

Data regarding the administration of intravenous albumin infusion to the patient was extracted from the database (itemid: 220863, 220861, 220862,220864, 30008, 30009, 30181, 44203, 40548, 43353, 42832, 45403, 43237).

### Data collection

The following data were extracted from the databases: age, gender, ethnicity, insurance, marital status, heart rate (bpm), respiratory rate (insp/min), systolic blood pressure (SBP, mmHg), diastolic blood pressure (DBP, mmHg), mean arterial pressure (MAP, mmHg), temperature (°C), cardiogenic shock, respiratory failure, diabetes, dyslipidemia, pleural effusion, Simplified Acute Physiology Score II (SAPSII), SOFA score, Glasgow Coma Score (GCS), Charlson comorbidity index (CCI), system inflammatory response syndrome(SIRS), oxygen saturation (SpO_2_, %), prothrombin time (PT), partial thromboplastin time (PTT), white blood cell (WBC, K/μL), platelet (K/μL), hemoglobin (g/dL), red cell distribution width (RDW, %), blood creatinine (mg/dL), blood urea nitrogen (BUN, mg/dL), total bilirubin (mg/dL), glucose (mg/dL), serum albumin (g/dL), aspartate aminotrasferase (AST, IU/L), alanine aminotransferase (ALT, IU/L), bicarbonate (mEq/L), sodium (mEq/L), potassium (mEq/L), chloride (mEq/L), magnesium (mg/dL), international normalized ratio (INR), mechanical ventilation, vasopressor, heparin, antibiotics, statins, and insulin. For patients with multiple ICU hospitalizations, only data from the initial ICU admission of each patient were included in this study.

### Statistical analysis

For continuous variables, skewness and kurtosis methods were employed to assess normality, while the Levene test was utilized to examine variance homogeneity. The continuous variables conforming to normal distribution were described using Mean and standard deviation [Mean (±SD)]. The t-test was applied for comparing groups with homogeneous variances, whereas the t’ test was used for comparing groups with heterogeneous variances. Non-normal continuous variables were presented as median and quartile intervals [M (Q₁, Q₃)], and inter-group comparisons were conducted using Wilcoxon rank-sum tests. Categorical variables are shown as the number of cases and composition ratio [n (%)]. Chi-square test or Fisher exact test was performed for between-group comparisons. Variables with missing values were imputed using multiple interpolation method. Multiple interpolation was carried out using R Package “mice” version 3.15.0 Index, with 5 iterations performed. The average value of the five interpolations was computed for continuous variables, while the modal number of the five interpolations was determined for categorical variables. S1 Table in S1 File shows the sensitivity analysis on the data sets before and after interpolation. *P* < 0.05 was regarded as statistically significant.

Confounding variables related to sepsis risk in this analysis were screened by univariate logistic regression analysis (S2 Table in S1 File, *P* < 0.05). The association between albumin infusion and sepsis risk of AP patients was evaluated using univariate and multivariate logistic regression models with odds ratio (OR) and 95% confidence interval (CI). Additional subgroup analysis by stratification to serum albumin (≤3.5g/L and>3.5g/L) [19] were also performed to explore whether the impact of albumin infusion on sepsis risk differed across these subsets. All analyses were performed with R software (version 4.2.3).

## Results

### Characteristics of study population

A total of 779 patients diagnosed with AP were included in this analysis, with a mean age of 58.3 (±17.80) years. Approximately 47.1% patients were female. Of the study cohort, 83 AP patients developed sepsis. Table 1 displays the variations in features between the sepsis group and non-sepsis group. AP patients in the sepsis group were more likely to have cardiogenic shock, respiratory failure, and use the vasopressor, antibiotics, insulin, and had higher values for SAPSII score, SOFA score, CCI, blood creatinine, total bilirubin than those in the non-sepsis groups. Detailed baseline information was given in Table 1.

**Table 1:** Characteristics description of the study population.

Variables	Overall (n = 779)	Non-sepsis group (n = 696)	Sepsis group (n = 83)	Statistics	*P*
Albumin infusion, yes, n (%)	70 (9.0)	64 (9.2)	6 (7.2)	χ² = 0.15	0.70
Age, years, Mean (±SD)	58.3 (±17.80)	58.1 (±18.1)	60.37 (±16.6)	t = −1.09	0.28
Gender, female, n (%)	367 (47.1)	327 (47.0)	40 (48.2)	χ² = 0.01	0.93
Ethnicity, n (%)				χ² = 2.62	0.45
White	501 (64.3)	450 (64.7)	51 (61.5)		
Black	71 (9.1)	62 (9.0)	9 (10.8)		
Other	73 (9.4)	68 (9.8)	5 (6.0)		
Unknown	134 (17.2)	116 (16.7)	18 (21.7)		
Insurance, n (%)				χ² = 0.54	0.76
Medicaid/Medicare/Government	427 (54.9)	379 (54.5)	48 (57.8)		
Private	178 (22.9)	159 (22.9)	19 (22.9)		
Other	174 (22.3)	158 (22.7)	16 (19.3)		
Marital status, n (%)				χ² = 3.21	0.36
Married	318 (40.8)	281 (40.3)	37 (44.6)		
Single	243 (31.2)	224 (32.2)	19 (22.9)		
Other	144 (18.5)	127 (18.3)	17 (20.5)		
Unknown	74 (9.5)	64 (9.2)	10 (12.1)		
Heart rate, bpm, Mean (±SD)	101.4 (±22.2)	101.2 (±21.8)	103.4(±24.5)	t = −0.86	0.39
SBP, mmHg, Mean (±SD)	128.2 (±26.0)	128.6 (±25.5)	125.1(±29.8)	t = 1.16	0.25
DBP, mmHg, Mean (±SD)	70.5 (±18.9)	70.6 (±18.7)	70.1(±20.6)	t = 0.21	0.83
MAP, mmHg, Mean (±SD)	89.8 (±19.0)	89.9 (±18.7)	88.4(±21.8)	t’ = 0.59	0.56
Respiratory rate, insp/min, Mean (±SD)	21.5 (±6.6)	21.3 (±6.4)	22.7(±7.4)	t = −1.87	0.061
Temperature, °C, n (%)				–	0.52
35 ~ 38	653 (83.9)	584 (83.9)	69 (83.13)		
< 35	35 (4.5)	33 (4.7)	2 (2.41)		
≥ 38	91 (11.7)	79 (11.4)	12 (14.46)		
Cardiogenic shock, n (%)				–	0.022
No	768 (98.6)	689 (99.0)	79 (95.2)		
Yes	11 (1.4)	7 (1.0)	4 (4.8)		
Respiratory failure, yes, n (%)	300 (38.5)	249 (35.8)	51 (61.5)	χ² = 19.57	<0.001
Diabetes, yes, n (%)	214 (27.5)	194 (27.9)	20 (24.1)	χ² = 0.36	0.55
Dyslipidemia, yes, n (%)	171 (22.0)	159 (22.8)	12 (14.5)	χ² = 2.58	0.11
Pleural effusion, yes, n (%)	129 (16.6)	118 (17.0)	11 (13.3)	χ² = 0.49	0.48
SAPSII, Mean (±SD)	38.1 (±16.5)	37.3 (±16.1)	45.4(±18.2)	t’ = −3.88	<0.001
SOFA, Mean (±SD)	6.0 (±4.1)	5.9 (±4.1)	7.6 (±4.1)	t = −3.69	<0.001
GCS, Mean (±SD)	12.4 (±3.9)	12.5 (±3.8)	11.1 (±4.4)	t’ = 2.99	0.0040
CCI, Mean (±SD)	3.5 (±2.7)	3.4 (±2.6)	4.4 (±3.3)	t’ = −2.43	0.017
SIRS, Mean (±SD)	3.2 (±0.8)	3.2 (±0.8)	3.2 (±0.8)	t = −0.24	0.81
SPO_2_, %, M (Q₁, Q₃)	97.0 (94.5, 99.0)	97.0 (95.0, 99.0)	96.0 (93.0, 98.5)	W = 33460.50	0.017
PT, second, M (Q₁, Q₃)	14.7 (13.4, 17.2)	14.7 (13.4, 17.2)	14.5 (13.3, 16.9)	W = 29839.50	0.62
PTT, second, M (Q₁, Q₃)	30.9 (27.2, 37.7)	30.9 (27.2, 37.7)	30.9 (26.9, 36.8)	W = 29846.50	0.62
WBC, K/uL, M (Q₁, Q₃)	12.9 (8.7, 18.5)	12.8 (8.5, 18.5)	14.0 (9.9, 19.5)	W = 26911.00	0.31
Platelet, K/uL, Mean (±SD)	214.1 (±127.6)	210.7 (±121.6)	243.4 (±167.9)	t’ = −1.72	0.088
Hemoglobin, g/dL, Mean (±SD)	11.3 (±2.2)	11.3 (±2.2)	11.3 (±2.4)	t = −0.14	0.89
RDW, %, Mean (±SD)	15.1 (±1.9)	15.0 (±1.9)	15.2 (±2.0)	t = −0.92	0.36
Blood creatinine, mg/dL, M (Q₁, Q₃)	1.0 (0.7, 2.0)	1.0 (0.7, 1.8)	1.2 (0.9, 2.7)	W = 24542.00	0.025
BUN, mg/dL, Mean (±SD)	30.1 (±25.7)	29.5 (±25.7)	34.5 (±25.2)	t = −1.68	0.094
Total bilirubin, mg/dL, M (Q₁, Q₃)	1.1 (0.6, 3.0)	1.1 (0.6, 2.9)	1.6 (0.8, 3.4)	W = 24634.00	0.028
Glucose, mg/dL, M (Q₁, Q₃)	127.0 (100.0, 174.5)	127.0 (100.0, 176.0)	130.0 (105.5, 162.5)	W = 28409.00	0.81
Serum albumin, g/dL, Mean (±SD)	2.9 (±0.6)	2.9 (±0.6)	2.8 (±0.6)	t = 1.40	0.16
AST, IU/L, M (Q₁, Q₃)	73.0 (36.0, 187.5)	76.0 (36.0, 192.6)	59.0 (34.5, 165.0)	W = 30347.50	0.45
ALT, IU/L, M (Q₁, Q₃)	59.0 (25.0, 158.5)	61.0 (26.0, 161.3)	43.0 (20.5, 99.5)	W = 32443.00	0.066
Bicarbonate, mEq/L, Mean (±SD)	20.9 (±5.7)	20.9 (±5.8)	20.8 (±5.5)	t = 0.24	0.81
Sodium, mEq/L, Mean (±SD)	138.3 (±5.8)	138.3 (±5.8)	138.4 (±5.4)	t = −0.13	0.90
Potassium, mEq/L, Mean (±SD)	4.1 (±0.9)	4.1 (±0.9)	4.3 (±1.1)	t = −1.58	0.12
Chloride, mEq/L, Mean (±SD)	105.3 (±7.3)	105.2 (±7.4)	105.9 (±6.4)	t = −0.77	0.44
Magnesium, mg/dL, Mean (±SD)	1.90 (±0.5)	1.89 (±0.5)	1.9 (±0.5)	t = −0.99	0.32
INR, M (Q₁, Q₃)	1.3 (1.2, 1.6)	1.30 (1.2, 1.6)	1.3 (1.2, 1.6)	W = 30289.50	0.47
Mechanical ventilation, yes, n (%)	456 (58.5)	404 (58.1)	52 (62.7)	χ² = 0.47	0.49
Vasopressor, yes, n (%)	254 (32.6)	210 (30.2)	44 (53.0)	χ² = 16.58	<0.001
Heparin, yes, n (%)	401 (51.5)	357 (51.3)	44 (53.0)	χ² = 0.03	0.86
Antibiotics, yes, n (%)	387 (49.7)	335 (48.1)	52 (62.7)	χ² = 5.69	0.017
Statins, yes, n (%)	91 (11.7)	83 (11.9)	8 (9.6)	χ² = 0.19	0.67
Insulin, yes, n (%)	540 (69.3)	472 (67.8)	68 (81.9)	χ² = 6.30	0.012

Abbreviations: SBP = systolic blood pressure; DBP = diastolic blood pressure; MAP = mean arterial pressure; SAPSII = Simplified Acute Physiology Score II; SOFA = Sequential Organ Failure Assessment; GCS = Glasgow coma scale Score; CCI = Charlson comorbidity index; SIRS = system inflammatory response syndrome; SpO_2_ = oxygen saturation; PT = prothrombin time; PTT = partial thromboplastin time; WBC = white blood cell; RDW = red cell distribution width; BUN = blood urea nitrogen; AST = aspartate aminotrasferase; ALT = alanine aminotransferase INR = international normalized ratio.

### Association between albumin infusion and sepsis

After performing univariate logistic regression analysis, we identified the confounding variables related to sepsis risk in this analysis, including cardiogenic shock, respiratory failure, SAPSII, SOFA, GCS, CCI, SPO_2_, platelet, total bilirubin, vasopressor, antibiotics, and insulin (*P* < 0.05). We developed and used univariate and multivariate regression models to assess the association between albumin infusion and sepsis risk of AP patients (Table 2). Model 1 was a crude model without covariate-adjusted; Model 2 was adjusted for all identified confounding variables, including cardiogenic shock, respiratory failure, SAPSII, SOFA, GCS, CCI, SPO2, platelet, total bilirubin, vasopressor, antibiotics, and insulin. Considering that the baseline levels of serum albumin and serum creatinine in patients may influence the correlation between albumin infusion and sepsis, we developed Model 3 that adjusted serum albumin, and blood creatinine based on the Model 2.

**Table 2:** Association between albumin infusion and sepsis.

Variables	Model 1		Model 2		Model 3	
	OR (95% CI)	*P*	OR (95% CI)	*P*	OR (95% CI)	*P*
Albumin infusion						
No	Ref		Ref		Ref	
Yes	0.77 (0.29-1.70)	0.56	0.37 (0.13-0.88)	0.039	0.37 (0.13-0.88)	0.037

OR=Odds ratio; CI=Confidence interval; Ref=reference.

Model 1 was a crude model without covariate-adjusted.

Model 2 was adjusted for cardiogenic shock, respiratory failure, Simplified Acute Physiology Score II (SAPSII), Sequential Organ Failure Assessment (SOFA), Glasgow coma scale Score (GCS), Charlson comorbidity index (CCI), oxygen saturation (SpO_2_), platelet, total bilirubin, vasopressor, antibiotics, and insulin.

Model 3 was adjusted for cardiogenic shock, respiratory failure, SAPSII, SOFA, GCS, CCI, SPO2, platelet, total bilirubin, vasopressor, antibiotics, insulin, serum albumin, and blood creatinine.

As shown in Table 2, the OR with 95% CI for the three models was presented. In the unadjusted model, there was no significant difference of albumin infusion with sepsis risk (*P* = 0.56). After adjusting cardiogenic shock, respiratory failure, SAPSII, SOFA, GCS, CCI, SPO_2_, platelet, total bilirubin, vasopressor, antibiotics, and insulin, albumin infusion was associated with decreased risk of sepsis in the AP patients (Model 2: adjusted OR=0.37, 95%CI = 0.13–0.88, *P* = 0.039). After further adjusting serum albumin, and blood creatinine based on the Model 2, the relationship between albumin infusion and sepsis risk remains unchanged, with an adjusted OR of 0.37 (95%CI = 0.13–0.88, *P* = 0.037).

### Subgroup analysis

To explore the relationship between albumin infusion and sepsis risk of AP patients stratified by serum albumin level, subgroup analyses were carried out. Prior to subgroup analysis, we conducted a comprehensive baseline comparison specifically for the AP patients with serum albumin ≤ 3.5g/L, as shown in S3 Table in S1 File. The standardized difference <0.2 indicates that all measured covariates are well balanced. In the Table 3, there was a negative association between the albumin infusion and sepsis risk in the AP patients with serum albumin ≤ 3.5g/L (adjusted OR=0.29, 95%CI = 0.08–0.77, *P* = 0.026).

**Table 3:** Subgroup analysis.

Subgroups	OR (95% CI)	*P*
Serum albumin≤3.5 g/L		
No	Ref	
Yes	0.29 (0.08-0.77)	0.026
Serum albumin>3.5 g/L		
No	Ref	
Yes	4.75 (0.28-85.85)	0.27

OR=Odds ratio; CI=Confidence interval; Ref=reference.

Adjusted for cardiogenic shock, respiratory failure, Simplified Acute Physiology Score II, Sequential Organ Failure Assessment, Glasgow coma scale Score, Charlson comorbidity index, oxygen saturation, platelet, total bilirubin, vasopressor, antibiotics, and insulin.

## Discussion

To our knowledge, this study is the first to investigate the association between albumin infusion and sepsis risk among patients with AP admitted to the ICU using a large national database. The finding indicated that albumin infusion was related to a decreased risk of sepsis after adjusting for confounding variables. Our results extended the application of the albumin infusion to the realm of sepsis, indicating its potential value for clinicians managing patients with AP admitted to the ICU.

Albumin is the most abundant protein in the plasma and serves as the primary protein to maintain fluid balance, transport molecules, and regulate pH [20]. Currently, albumin therapy was considered to be utilized for addressing hypoproteinemia and conducting fluid resuscitation in critically ill patients, such as those with septicemia and cirrhosis [21,22]. The impact of albumin infusion in critically ill patients, however, remains a subject of controversy. In the meta-analysis of randomized clinical trials, albumin use for resuscitation was found to be significantly associated with a reduction in 90-day mortality among septic shock patients, with an OR of 0.81 (95% CI: 0.67–0.97), and might slightly improve the outcome of severe sepsis patients when compared to crystalloid [23]. Given that some patients received albumin infusions and others did not, we have divided albumin administration into the following three scenarios: Protocol-driven hypoalbuminemia (albumin <3.5 g/dL), hemodynamic resuscitation (MAP < 65 mmHg with lactate >4), rescue therapy (refractory to ≥30 mL/kg crystalloids). We found that sepsis risk reduction remained significant across all strata (adjusted OR=0.62–0.71, p < 0.05 for each). The findings of a study conducted on critically ill patients with traumatic brain injury indicated that fluid resuscitation using albumin was associated with higher mortality rates compared to resuscitation with saline solution [24]. In addition, albumin infusion may increase the in-hospital mortality, as well as the length of stay in the ICU and the hospital among ICU patients with congestive heart failure -hypoalbuminemia overlap [12]. Although published study did assess the role of albumin in the prognosis of disease, limited attention has been given to the impact of albumin perfusion on the susceptibility to sepsis in patients with AP. In this regard, our research fills this research gap, and provides evidence that albumin infusion was likely to decrease the risk of sepsis of AP patients. However, the findings of Ma et al [25] contradicted our results, as they highlighted that albumin infusion did not confer any benefits on the in-hospital prognosis of AP patients and was instead associated with prolonged hospital and ICU stays. We performed time-varying analyses to examine the trajectory of albumin changes, as shown in S4 Table in S1 File. The table shows that there is a significant correlation between albumin level changes and the probability of sepsis. Compared with simple drug administration, albumin dynamics may be more clinically significant, and the effect of infusion and natural recovery seem to be synergistic. The initial serum albumin level of patients may exert an influence on the outcomes of clinical practice studies. Therefore, we also conducted subgroup analysis by stratifying according to serum albumin levels, which revealed that AP patients with serum albumin ≤ 3.5g/L who received albumin infusions was associated with lower sepsis risk. These findings suggested that albumin infusion was particularly beneficial for critically ill AP patients with hypoalbuminemia.

The possible underlying mechanism regarding the benefit of albumin infusion on the risk of sepsis in AP patients might be elucidated as follows: (1) AP, characterized by a proinflammatory response, may lead to systemic inflammatory response syndrome and organ failure [26]. (2)Albumin serves not only as an indicator of nutritional status, but also as a marker for anti-inflammatory response [27]. (3) When the body experiences the stress response due to acute and critical illness, there is a fluctuation in platelet count, which can significantly impact sepsis [2,28]. (4) Albumin acts as an antioxidant that scavenges free radicals and plays a crucial role in inhibiting platelet aggregation and maintaining capillary permeability [29]. Further studies are necessary to explore the detailed mechanism of albumin infusion on the risk of sepsis in AP patients.

It is important to acknowledge the limitations of this study. Firstly, this was a single-center retrospective cohort study, we were unable to avoid selection bias. Secondly, the dosage of albumin infusion could not be definitively determined due to limitations in the MIMIC-III and MIMIC-IV databases, which impeded our ability to assess the impact of albumin infusion. Thus, clinicians should be cautious about albumin infusion for AP patients in the clinical practice. However, MIMIC-III patients were treated under earlier resuscitation paradigms, where albumin was often reserved for more severe cases. We believe that fluid management practices, including the use of albumin, have significantly changed during this period. Therefore, we need to conduct stratified analyses of different database versions in future trials and perform sensitivity analyses with time-dependent adjustments. Lastly, given that the study exclusively involved participants from United States, the generalizability of the findings to other countries may be limited. Our study needs to be corroborated and validated through prospective multicenter studies involving larger cohort size in the future.

## Conclusion

In this study, we found an association between albumin administration and a lower risk of sepsis in AP patients, which persisted after multivariate adjustment. This suggests that albumin infusion may have unique potential benefits for this population.

## Supporting information

S1 File **Table 1.** Sensitivity analysis on the data sets before and after interpolation. **Table 2.** The screening of confounding variables. **Table 3.** Subgroup Balance Analysis. **Table 4.** Albumin Change Trajectories.
(DOCX)
