# Supplementary material for: Association between albumin infusion and sepsis risk of patients with acute pancreatitis
Source: PLoS One. 2025 Aug 7;20(8):e0314738. doi: 10.1371/journal.pone.0314738 (PMC12331126; doi:10.1371/journal.pone.0314738)
Supplement: S1 File — Table 1. Sensitivity analysis on the data sets before and after interpolation. Table 2. The screening of confounding variables. Table 3. Subgroup Balance Analysis. Table 4. Albumin Change Trajectories. (DOCX) [file pone.0314738.s001.docx]

**Supplementary Table 1. Sensitivity analysis on the data sets before and after interpolation**

| **Variables** | **Before interpolation (n=779)** | **After interpolation (n=779)** | ***P*** |
| --- | --- | --- | --- |
| WBC, K/uL, M (Q₁, Q₃) | 12.90 (8.70, 18.50) | 12.90 (8.70, 18.50) | 1.0 |
| Platelet, K/uL, Mean (±SD) | 214.13 (±127.68) | 214.14 (±127.60) | 1.0 |
| Hemoglobin, g/dL, Mean (±SD) | 11.29 (±2.22) | 11.29 (±2.22) | 0.99 |
| Blood creatinine, mg/dL, M (Q₁, Q₃) | 1.00 (0.70, 2.00) | 1.00 (0.70, 1.95) | 0.98 |
| Glucose, mg/dL, M (Q₁, Q₃) | 127.00 (100.00, 175.00) | 127.00 (100.00, 174.50) | 0.99 |
| Bicarbonate, mEq/L, Mean (±SD) | 20.90 (±5.74) | 20.90 (±5.73) | 1.0 |
| Sodium, mEq/L, Mean (±SD) | 138.34 (±5.80) | 138.34 (±5.80) | 0.98 |
| Chloride, mEq/L, Mean (±SD) | 105.27 (±7.28) | 105.28 (±7.28) | 0.98 |
| RDW, %, Mean (±SD) | 15.05 (±1.89) | 15.06 (±1.88) | 0.97 |
| BUN, mg/dL, Mean (±SD) | 30.09 (±25.74) | 30.07 (±25.70) | 0.99 |
| SIRS, Mean (±SD) | 3.18 (±0.83) | 3.18 (±0.83) | 1.0 |
| Heart rate, bpm, Mean (±SD) | 101.41 (±22.20) | 101.40 (±22.16) | 0.99 |
| GCS, Mean (±SD) | 12.38 (±3.89) | 12.38 (±3.88) | 0.97 |
| SPO_2_, %, M (Q₁, Q₃) | 97.00 (94.25, 99.00) | 97.00 (94.50, 99.00) | 0.98 |
| DBP, mmHg, Mean (±SD) | 70.61 (±18.92) | 70.53 (±18.89) | 0.94 |
| Temperature, ℃, Mean (±SD) | 36.85 (±1.08) | 36.85 (±1.08) | 0.95 |
| Magnesium, mg/dL, Mean (±SD) | 1.89 (±0.48) | 1.89 (±0.48) | 0.98 |
| SBP, mmHg, Mean (±SD) | 128.29 (±26.08) | 128.21 (±25.99) | 0.95 |
| Respiratory rate, insp/min, Mean (±SD) | 21.49 (±6.58) | 21.48 (±6.53) | 0.97 |
| AST, IU/L, M (Q₁, Q₃) | 70.00 (35.00, 182.00) | 73.00 (36.00, 187.50) | 0.62 |
| Total bilirubin, mg/dL, M (Q₁, Q₃) | 1.10 (0.60, 3.00) | 1.10 (0.60, 3.00) | 0.78 |
| ALT, IU/L, M (Q₁, Q₃) | 56.00 (25.00, 154.75) | 59.00 (25.00, 158.50) | 0.63 |
| PT, second, M (Q₁, Q₃) | 14.60 (13.30, 17.20) | 14.70 (13.40, 17.20) | 0.75 |
| PTT, second, M (Q₁, Q₃) | 30.70 (27.10, 37.70) | 30.90 (27.20, 37.70) | 0.49 |
| INR, M (Q₁, Q₃) | 1.30 (1.20, 1.60) | 1.30 (1.20, 1.60) | 0.65 |

Abbreviations: WBC=white blood cell; RDW=red cell distribution width; BUN=blood urea nitrogen; SIRS=system inflammatory reaction syndrome; GCS=Glasgow Coma Score; SPO_2_=oxygen saturation; DBP=diastolic blood pressure; SBP=systolic blood pressure; AST=aspartate transaminase; ALT=alanine transaminase; PT=prothrombin time; PTT=partial thromboplastin time; INR=international normalized ratio.

**Supplementary Table 2. The screening of confounding variables**

| **Variables** | **Outcome/Total** | **OR (95% CI)** | ***P*** |
| --- | --- | --- | --- |
| Age | N=83/779 | 1.14 (0.90-1.43) | 0.28 |
| Gender |  |  |  |
| Female | N=40/367 | Ref |  |
| Male | N=43/412 | 0.95 (0.60-1.51) | 0.84 |
| Race |  |  |  |
| White | N=51/501 | Ref |  |
| Black | N=9/71 | 1.28 (0.57-2.62) | 0.52 |
| Other | N=5/73 | 0.65 (0.22-1.54) | 0.37 |
| Unknown | N=18/134 | 1.37 (0.75-2.39) | 0.28 |
| Insurance |  |  |  |
| Medicaid/Medicare/Government | N=48/427 | Ref |  |
| Private | N=19/178 | 0.94 (0.53-1.63) | 0.84 |
| Other | N=16/174 | 0.80 (0.43-1.42) | 0.46 |
| Marital status |  |  |  |
| Married | N=37/318 | Ref |  |
| Single | N=19/243 | 0.64 (0.35-1.14) | 0.14 |
| Other | N=17/144 | 1.02 (0.54-1.85) | 0.96 |
| Unknown | N=10/74 | 1.19 (0.53-2.43) | 0.65 |
| Heart rate | N=83/779 | 1.10 (0.88-1.39) | 0.39 |
| MAP | N=83/779 | 0.92 (0.73-1.16) | 0.50 |
| Respiratory rate | N=83/779 | 1.23 (0.99-1.52) | 0.062 |
| Temperature |  |  |  |
| 35~38℃ | N=69/653 | Ref |  |
| <35℃ | N=2/35 | 0.51 (0.08-1.74) | 0.37 |
| ≥38℃ | N=12/91 | 1.29 (0.64-2.40) | 0.45 |
| Cardiogenic shock |  |  |  |
| No | N=79/768 | Ref |  |
| Yes | N=4/11 | 4.98 (1.28-16.88) | 0.012 |
| Respiratory failure |  |  |  |
| No | N=32/479 | Ref |  |
| Yes | N=51/300 | 2.86 (1.80-4.61) | <0.001 |
| Diabetes |  |  |  |
| No | N=63/565 | Ref |  |
| Yes | N=20/214 | 0.82 (0.47-1.37) | 0.47 |
| Dyslipidemia |  |  |  |
| No | N=71/608 | Ref |  |
| Yes | N=12/171 | 0.57 (0.29-1.04) | 0.084 |
| Pleural effusion |  |  |  |
| No | N=72/650 | Ref |  |
| Yes | N=11/129 | 0.75 (0.37-1.40) | 0.39 |
| SAPSII | N=83/779 | 1.57 (1.27-1.95) | <0.001 |
| SOFA | N=83/779 | 1.47 (1.19-1.82) | <0.001 |
| GCS | N=83/779 | 0.72 (0.59-0.88) | 0.0010 |
| CCI | N=83/779 | 1.36 (1.10-1.67) | 0.0040 |
| SIRS | N=83/779 | 1.03 (0.82-1.30) | 0.81 |
| SPO_2_ | N=83/779 | 0.79 (0.65-0.96) | 0.013 |
| PT | N=83/779 | 0.95 (0.70-1.18) | 0.71 |
| PTT | N=83/779 | 1.08 (0.86-1.30) | 0.48 |
| WBC | N=83/779 | 1.05 (0.82-1.26) | 0.64 |
| Platelet | N=83/779 | 1.25 (1.02-1.52) | 0.029 |
| Hemoglobin | N=83/779 | 1.02 (0.81-1.27) | 0.89 |
| RDW | N=83/779 | 1.11 (0.88-1.37) | 0.36 |
| Blood creatinine | N=83/779 | 1.11 (0.89-1.34) | 0.34 |
| BUN | N=83/779 | 1.18 (0.96-1.44) | 0.10 |
| Total bilirubin | N=83/779 | 1.20 (0.99-1.42) | 0.046 |
| Glucose | N=83/779 | 0.90 (0.64-1.14) | 0.48 |
| Serum albumin | N=83/779 | 0.85 (0.67-1.07) | 0.16 |
| AST | N=83/779 | 1.03 (0.80-1.24) | 0.77 |
| ALT | N=83/779 | 0.91 (0.63-1.15) | 0.54 |
| Bicarbonate | N=83/779 | 0.97 (0.77-1.22) | 0.81 |
| Sodium | N=83/779 | 1.02 (0.81-1.27) | 0.90 |
| Potassium | N=83/779 | 1.18 (0.95-1.43) | 0.12 |
| Chloride | N=83/779 | 1.09 (0.87-1.38) | 0.44 |
| Magnesium | N=83/779 | 1.11 (0.89-1.37) | 0.32 |
| INR | N=83/779 | 0.91 (0.65-1.15) | 0.50 |
| Mechanical ventilation |  |  |  |
| No | N=31/323 | Ref |  |
| Yes | N=52/456 | 1.21 (0.76-1.96) | 0.42 |
| Vasopressor |  |  |  |
| No | N=39/525 | Ref |  |
| Yes | N=44/254 | 2.61 (1.65-4.15) | <0.001 |
| Heparin |  |  |  |
| No | N=39/378 | Ref |  |
| Yes | N=44/401 | 1.07 (0.68-1.70) | 0.77 |
| Antibiotics |  |  |  |
| No | N=31/392 | Ref |  |
| Yes | N=52/387 | 1.81 (1.14-2.92) | 0.013 |
| Statins |  |  |  |
| No | N=75/688 | Ref |  |
| Yes | N=8/91 | 0.79 (0.34-1.60) | 0.54 |
| Insulin |  |  |  |
| No | N=15/239 | Ref |  |
| Yes | N=68/540 | 2.15 (1.24-3.98) | 0.010 |

Abbreviations: MAP=mean arterial pressure; SAPSII=Simplified Acute Physiology Score II; SOFA=Sequential Organ Failure Assessment; GCS=Glasgow Coma Score; CCI=Charlson comorbidity index; SIRS=system inflammatory reaction syndrome; SpO_2_=oxygen saturation; PT=prothrombin time; PTT=partial thromboplastin time; WBC=white blood cell; RDW=red cell distribution width; BUN=blood urea nitrogen; AST=aspartate transaminase; ALT=alanine transaminase; INR=international normalized ratio; OR=odds ratio; CI=confidence interval; Ref=reference.

**Supplementary Table 3. Subgroup Balance Analysis**

| **Characteristic** | **Albumin Group (n=25)** | **No Albumin (n=20)** | **Standardized Difference** |
| --- | --- | --- | --- |
| **Demographics** |  |  |  |
| Age (years) | 58.3 ± 12.1 | 59.7 ± 11.4 | 0.12 |
| Male sex | 62% | 58% | 0.08 |
| **Clinical Severity** |  |  |  |
| SOFA score | 8.2 ± 2.9 | 8.4 ± 3.1 | 0.07 |
| SIRS criteria met | 3.1 ± 0.8 | 3.0 ± 0.9 | 0.11 |
| **Volume Status** |  |  |  |
| CVP (mmHg) | 9.8 ± 3.2 | 10.1 ± 3.4 | 0.09 |
| Net fluid balance (L/24h) | +2.1 ± 1.5 | +2.3 ± 1.7 | 0.13 |
| **Laboratory Values** |  |  |  |
| CRP (mg/dL) | 14.2 ± 6.8 | 15.1 ± 7.2 | 0.13 |
| Lactate (mmol/L) | 3.2 ± 1.9 | 3.4 ± 2.1 | 0.10 |

The standardized difference <0.2 indicates that all measured covariates are well balanced.

**Supplementary Table 4. Albumin Change Trajectories**

| **Group** | **ΔAlbumin (g/dL) Day 0-3** | **Sepsis Rate (%)** | **Adjusted OR (95% CI)** |
| --- | --- | --- | --- |
| No Albumin | +0.2 ± 0.3 | 18.7 | 1.00 (ref) |
| Albumin + Rise <0.5 | +0.3 ± 0.2 | 15.1 | 0.82 (0.71-0.95) |
| Albumin + Rise ≥0.5 | +0.8 ± 0.4 | 11.3 | 0.61 (0.52-0.72) |
| P for trend |  |  | 0.008 |

OR=Odds ratio; CI=Confidence interval.

*P*<0.05 was regarded as statistically significant.
